# Case Report: Significant Efficacy of Pyrotinib in the Treatment of Extensive Human Epidermal Growth Factor Receptor 2-Positive Breast Cancer Cutaneous Metastases: A Report of Five Cases

**DOI:** 10.3389/fonc.2021.729212

**Published:** 2021-12-16

**Authors:** Nan Wang, Lin Li, Youyi Xiong, Jiangrui Chi, Xinwei Liu, Chaochao Zhong, Fang Wang, Yuanting Gu

**Affiliations:** ^1^ Department of Breast Surgery, The First Affiliated Hospital of Zhengzhou University, Zhengzhou, China; ^2^ Department of Plastic Surgery, The First Affiliated Hospital of Zhengzhou University, Zhengzhou, China

**Keywords:** HER2-positive breast cancer, pyrotinib, cutaneous metastases, tyrosine kinase inhibitors (TKIs), case report

## Abstract

**Background:**

Breast cancer (BC) is the most common tumor to develop cutaneous metastases. Most BCs with cutaneous metastasis are human epidermal growth factor receptor 2 (HER2)-positive subtypes. Although the molecular mechanisms of breast cancer metastasis to different sites and the corresponding treatment methods are areas of in-depth research, there are few studies on cutaneous metastasis.

**Case Presentation:**

Five HER2-positive BC patients with extensive cutaneous metastases were treated with a regimen containing pyrotinib, a novel small-molecule tyrosine kinase inhibitor that irreversibly blocks epidermal growth factor receptor (EGFR), HER2, and human epidermal growth factor receptor 4 (HER4), then their cutaneous metastases quickly resolved at an astonishing speed and their condition was well controlled during the follow-up period.

**Conclusions:**

This case series reports the significant therapeutic effect of pyrotinib on cutaneous metastases of HER2-positive BC for the first time. Based on this, we recommend that pyrotinib can be used as a supplement to trastuzumab for HER2-positive BC patients with cutaneous metastases. In addition, we should consider that the pan-inhibitory effect of pyrotinib on EGFR, HER2, and HER4 may provide a dual therapeutic effect against HER2 and mucin 1.

## Introduction

Breast cancer (BC) is the most common primary tumor in cutaneous metastatic malignancies, and the HER2-positive subtype was more common than other subtypes in cutaneous metastasis of BC (8.3%) (1–4). Tumor tissue necrosis caused by the conventional treatment of cutaneous metastases of BC often causes temporary aggravation of cutaneous symptoms, which leads to increased subjective pain and misjudgment of the treatment effect (5). For HER2-positive BC patients, the use of anti-HER2 monoclonal antibody (mAb) is the standard treatment. However, related studies have found the existence of immune privilege in the skin (6). Immune privilege can weaken the antibody-dependent cell-mediated cytotoxicity (ADCC), which is an important factor related to the efficacy of anti-HER2 mAbs (7–10). This suggests that anti-HER2 mAbs may not be effective in mediating an effective therapeutic effect in the cutaneous metastases of HER2-positive BC. There have been reports of some cases of cutaneous progression during or after treatment with anti-HER2 mAbs (11–14). Pyrotinib is an oral, irreversible pan-ErbB receptor tyrosine kinase inhibitor (TKI), which has anti-epidermal growth factor receptor (EGFR)/HER1, HER2, and HER4 activity (15). There is evidence that HER2 targeting TKIs can enhance the ADCC response and thus act in synergy with anti-HER2 mAbs in the treatment of BC (16, 17). There have been clinical reports about the therapeutic effect of pyrotinib in patients with metastatic breast cancer (MBC) who have failed trastuzumab and pertuzumab treatment (18–23), and pyrotinib has also been found to have a good therapeutic effect on other types of HER2-positive advanced malignancies (24–30).

Here we report five cases of HER2-positive BC patients with severe cutaneous metastases. All patients received treatments containing pyrotinib and got a rapid treatment response with a remarkable disease-free time. This finding suggests that pyrotinib has good potential as a treatment option for patients with BC cutaneous metastases.

## Case Presentation

### Case 1

**Figure 1:**
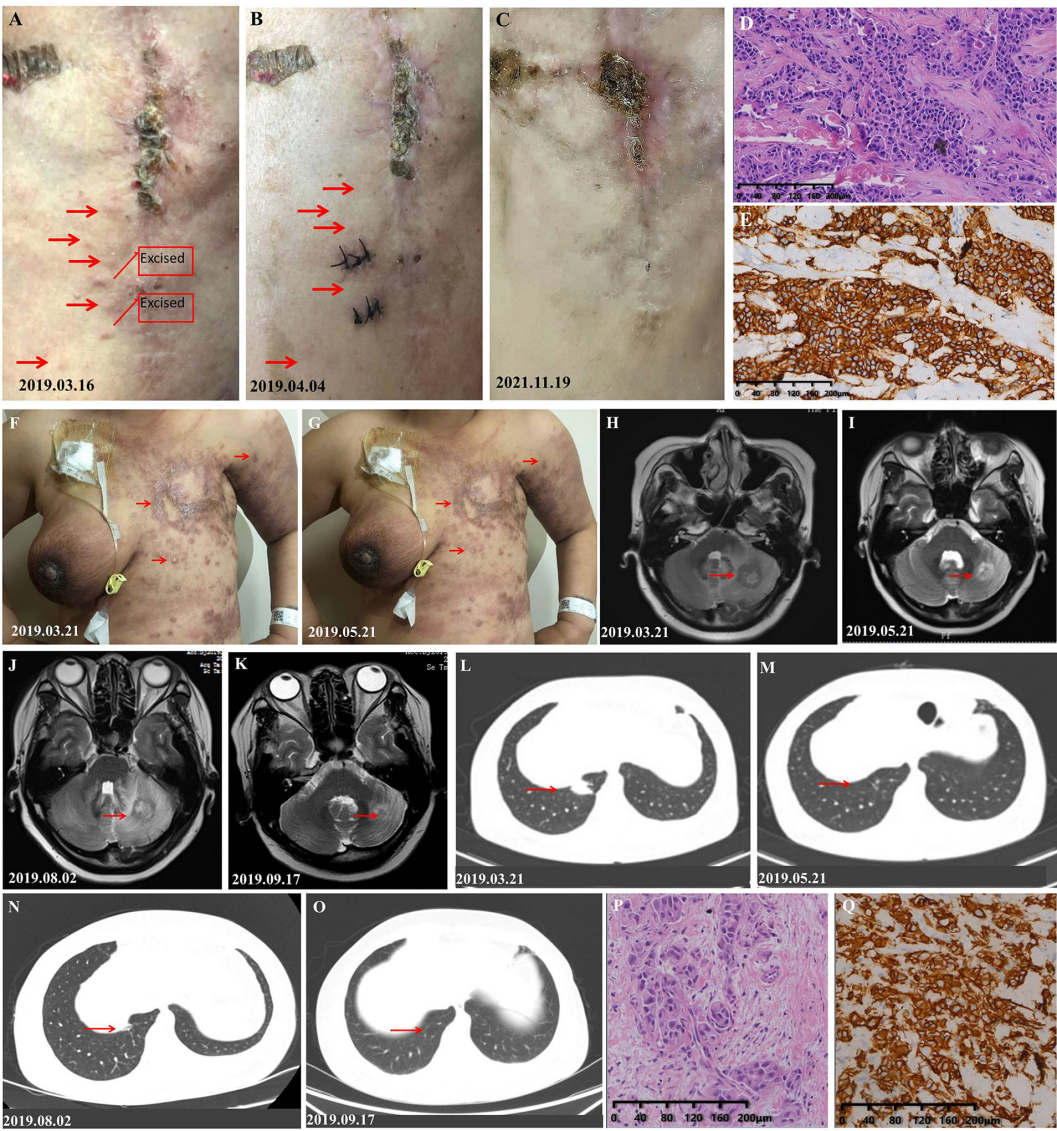
Response of the metastases to treatment of the patients in cases 1 and 2. **(A)** Photograph taken before treatment: The skin on the left chest wall is diffused with painless hard nodules, accompanied by redness and swelling. **(B)** Photograph taken after one cycle of treatment, at which point, the redness and hard nodules of the cutaneous had obviously subsided. **(C)** Photograph taken on the latest follow-up date showing hypertrophic scars formed by repeated shedding and new scabs. **(D)** H&E staining image of tumor samples taken from the left chest wall of the patient in case 1. **(E)** Very strong immunohistochemical staining (IHC 3+) of HER2 was observed in the tumor samples taken from the left chest wall of the patient in case 1. **(F)** Photograph of the patient in case 2 taken before treatment: The skin on the left chest wall is diffused with hard nodules, and the skin around the edge of the lesion is red and swollen. **(G)** Photograph taken after two cycles of treatment, at which point the redness and hard nodules of the cutaneous had obviously subsided. **(H)** Photograph showing the size of the pulmonary lesion before the initial treatment. **(I)** Photograph showing the shrinkage of the pulmonary lesion after two courses of treatment. **(J)** Photograph showing the progression of pulmonary lesion after she had stopped pyrotinib. **(K)** Photograph which shows that the pulmonary lesion was controlled again after pyrotinib was resumed. **(L)** Photograph showing the size of the intracranial lesion before the initial treatment. **(M)** Photograph showing the shrinkage of the intracranial lesion after two courses of treatment. **(N)** Photograph showing the progression of the intracranial lesion after she had stopped pyrotinib. **(O)** Photograph showing that the intracranial lesion was controlled again after pyrotinib was resumed. **(P)** H&E staining of the 2017 breast tissue specimen taken from the patient in case 2 (she refused to take a new tissue for pathological examination.) **(Q)** Very strong immunohistochemical staining (IHC 3+) of HER2 was observed in the tumor samples of the patient in case 2.

A 57-year-old woman who suffered from pathology-confirmed chest wall BC metastasis for more than 5 months was admitted to our hospital for evaluation on March 9, 2019. She was diagnosed with infiltrating ductal carcinoma of the left breast cancer and had undergone mastectomy on December 3, 2015. The immunohistochemistry indicated estrogen receptor negative (ER−), progesterone receptor negative (PR−), HER-2(3+), and Ki-67 (approximately 50% +). She was first diagnosed with chest wall metastasis of BC in April 2018 and had received multiple regimens previously, including anthracyclines, paclitaxel-based chemotherapeutics, and trastuzumab (**Supplementary Figure S1**). On admission, the patient appeared to be in a good clinical condition, and the performance status (PS) was 1. She had no smoking and drinking habits and no other chronic diseases. The physical examination results showed painless hard nodules, accompanied by redness and swelling, on the left chest wall around the incision made for radical breast cancer surgery (**Figure 1A**). The results of a further examination including CT and MRI showed that she had no metastasis to other organs. We removed a mass from her left chest wall for histopathological examination and found that her breast cancer had metastases to the chest wall (**Figure 1D**). And the immunohistochemical results revealed positive staining for HER2 (**Figure 1E**). We performed genetic testing and found that she had copy number amplification of *ERBB2* (8.4-fold higher than normal), a p.R342 non-significant mutation in exon 10 of *TP53* (abundance, 46.9%), and *PIK3CA* exon 1 p.E110del mutation (28.7%, abundance). Based on the clinical history, pathological findings, and Clinical Practice Guidelines in Oncology (NCCN) (V2019.1), we confirmed that she had a stage IV breast cancer with possible cross-resistance to pertuzumab and ado-trastuzumab emtansine (TDM-1). We formulated the “pyrotinib, capecitabine, and trastuzumab” program for her, and started her treatment on March 16, 2019. She was discharged from the hospital on March 19, 2019 and took oral medication at home as planned. She returned to the hospital on April 4, 2019 to prepare for further treatment. Then, we found that the red and swollen area on her chest wall had completely disappeared (**Figure 1B**). Her cancer cells remained active but could always be inhibited, so the scab of her left chest wall continued to fall off and regenerate (**Figure 1C**). Her last follow-up was November 17, 2021, by which point the disease control time had reached 20 months.

### Case 2

A 41-year-old woman with blurred vision was admitted to our department on March 19, 2019. On physical examination, diffuse and painless indurated nodules were found on the cutaneous of the left chest wall and abdominal wall, which were accompanied by redness and ulcer formation. Cutaneous manifestations of inflammatory breast cancer were also observed on the right breast (**Figure 1F**). At the time of admission, her brain, lung, and cutaneous metastases of breast cancer had a high tumor burden (**Figures 1H, L**), and her PS was 2. She had no smoking and drinking habits and no other chronic diseases such as hypertension. She was diagnosed with HER2 (+) BC (**Figures 1P, Q**) and treated at our hospital in February 2017; the specific treatment events and disease progressions are shown in **Supplementary Figure S2**. The disease progression during the course of trastuzumab-targeted therapy and taxane drugs suggested that she had primary resistance to trastuzumab and taxane drugs. We confirmed that she had a stage IV breast cancer according to the NCCN Guidelines (V2019.1). We treated her with vinorelbine combined with trastuzumab and pyrotinib regimen, which achieved good results (**Figures 1G, I, M**). However, she decided to discontinue pyrotinib after her cutaneous lesions improved, which led to the progression of her intracranial and pulmonary lesions (**Figures 1J, N**). Then, she resumed the standard dosage of pyrotinib and achieved good results again (**Figures 1K, O**). Eventually, she refused further treatment and left our hospital. In a telephone follow-up, she said that the drugs, except pyrotinib, had been discontinued. If she felt a headache, she would take pyrotinib orally until the headache disappeared and then stop taking it again. The last telephone follow-up was on March 3, 2020, when she had received more than 11 months of maintenance treatment. After that, she declined our follow-up.

### Case 3

**Figure 2:**
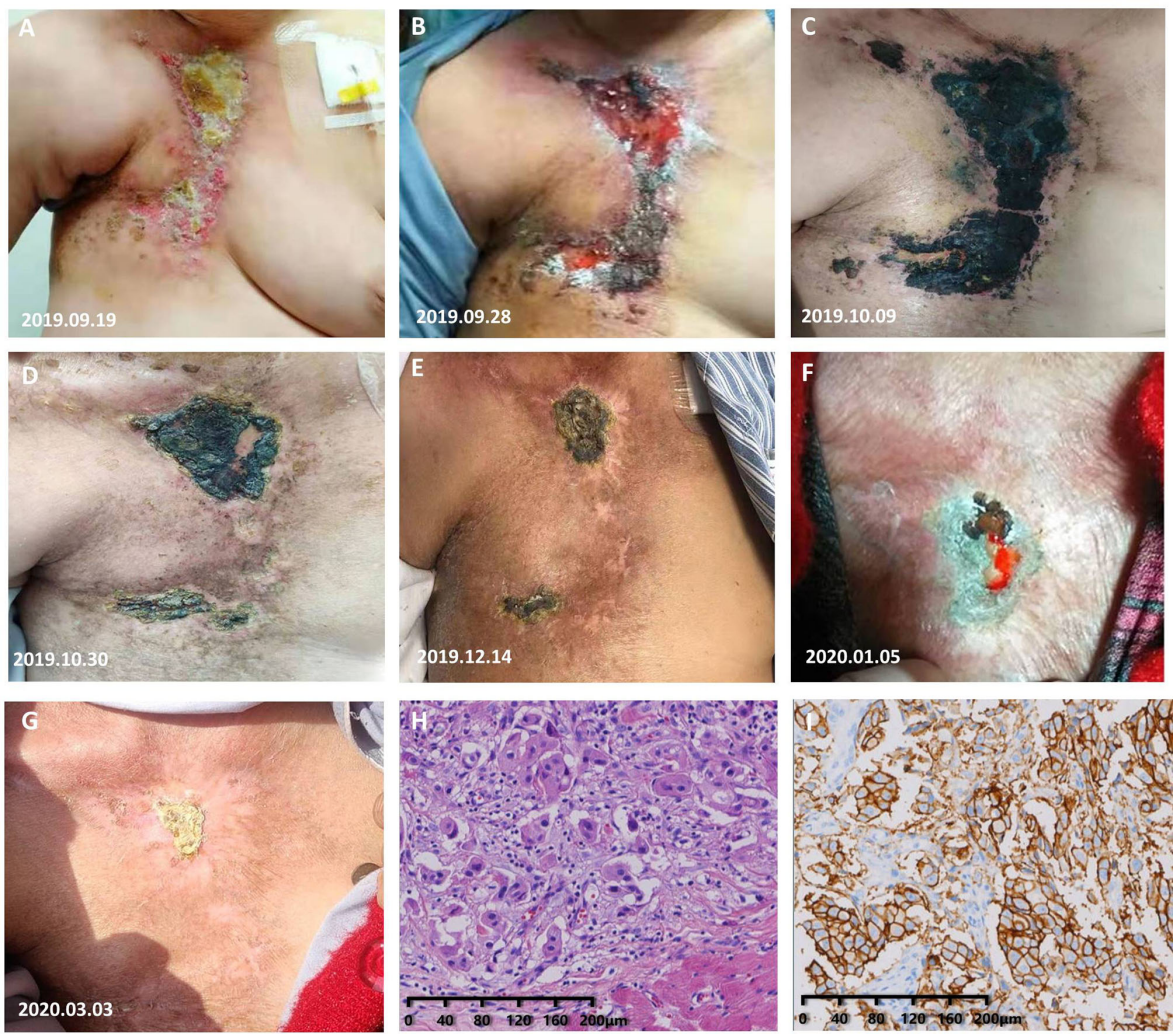
Response of the tumor metastases to treatment. **(A)** Photograph taken before treatment: The skin on the left chest wall was diffused with nodules and ulcerated skin on the surface, accompanied by redness and swelling. **(B)** Photograph taken after 8 days of treatment: The redness and hard nodules of the skin had obviously subsided. **(C–E)** The skin lesions shrank quickly. **(F)** The ulcer on the chest wall continued to progress after she chose to reduce the dose of pyrotinib. **(G)** The skin lesions shrank rapidly after she resumed the standard dose of pyrotinib. **(H)** H&E staining image of tumor samples taken from the chest wall of the patient in case 3. **(I)** Very strong immunohistochemical staining (IHC 3+) of HER2 was observed in the tumor samples taken from the left chest wall of the patient in case 3.

A 64-year-old woman was admitted to the hospital for evaluation on September 11, 2019. The physical examination results revealed an extensive distribution of elevated cutaneous nodules with ulceration on the right chest wall. The lesion area was about 10 cm × 20 cm and extended to the neck (**Figure 2A**), and her PS was 2. She had no smoking and drinking habits and no chronic diseases. The diagnosis and treatment events in the past are shown in **Supplementary Figure S3**. The imaging examination results showed that her BC did not metastasize to distant organs. The pathological result of the chest wall nodule was HER2-positive BC metastasis (**Figures 2H, I** **)**. We confirmed that she had a stage IV BC according to the NCCN Guidelines (V2019.1) and treated her with docetaxel combined with trastuzumab and pyrotinib regimen on September 19, 2019 (the usage and dosage details are shown in **Supplementary Figure S3K**). After 8 days of treatment, the tumor lesions on her cutaneous showed scabs in the ulcer area and shrinkage of the lesion area (**Figure 2B**). After continuous treatment, the condition of the patient gradually improved (**Figures 2C–E**). Then, she reduced her dose of pyrotinib in half on the 64th day of treatment. After 30 days of dose reduction, the ulcer on her chest wall had progressed (**Figure 2F**). Then, she restored the dosage of pyrotinib to 400 mg/day and subsequently had significant therapeutic effects (**Figure 2G** **)**. After completing six cycles of docetaxel, the patient continued the maintenance treatment with trastuzumab combined with pyrotinib and capecitabine. The last follow-up was March 3, 2020. At this time, her progression-free survival had been maintained for 6 months. The patient then declined the planned telephone follow-up.

### Case 4

**Figure 3:**
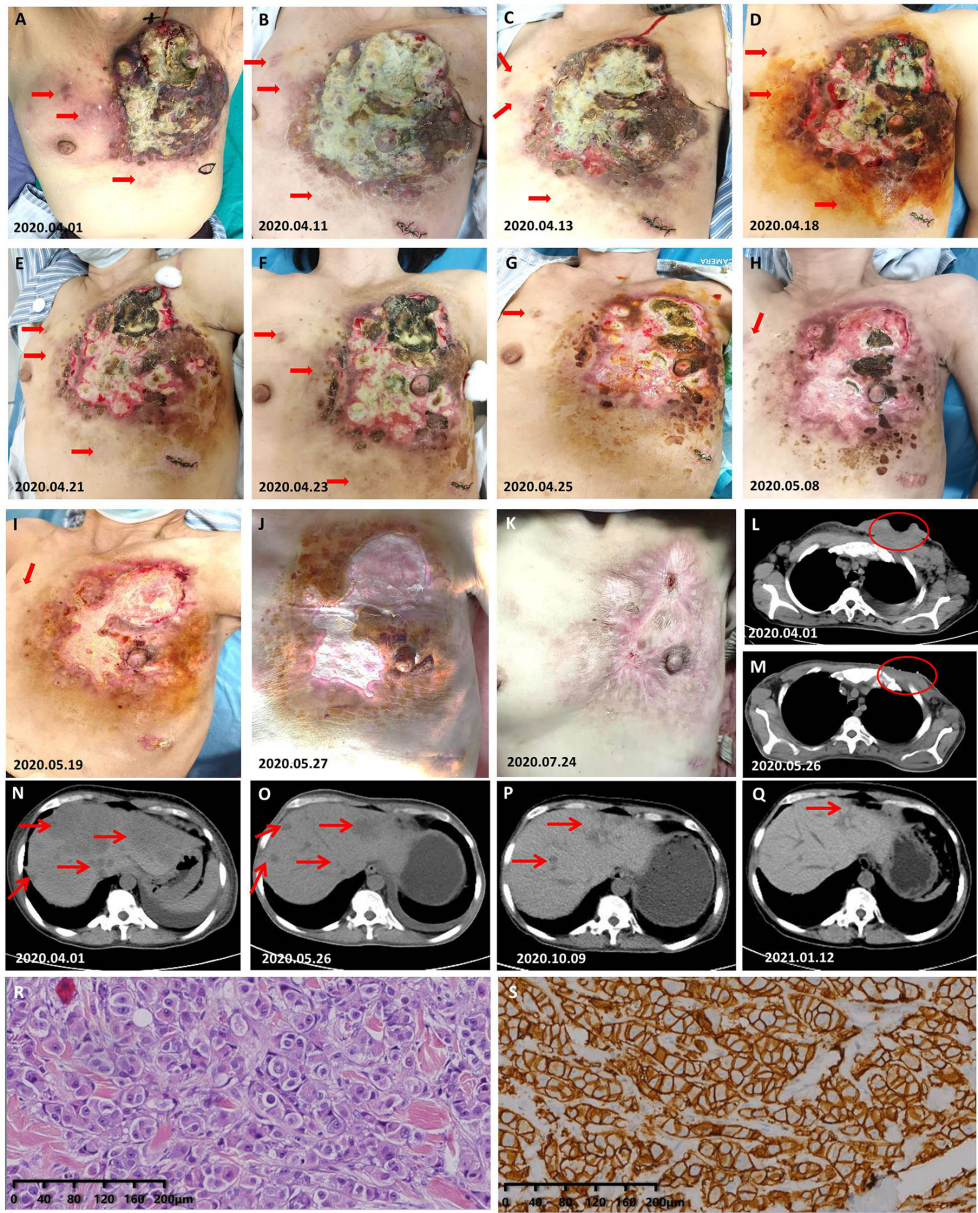
Response of the cutaneous and liver tumor metastases to the treatment. **(A)** Photograph taken on the day of pathological biopsy. The skin nodules marked are the tissues to be removed. **(B)** On the 3rd day after she received low-dose capecitabine and standard-dose pyrotinib; albumin paclitaxel and trastuzumab was increased at this point. **(C–J)** Gross changes in the tumor over time. **(K)** The chest wall that was once covered by a huge mass is now completely covered by skin. **(L)** Prior to primary treatment, the chest wall CT showed considerable soft tissue with necrotic cavities. **(M)** At this time, the chest wall CT showed that the tumor had disappeared and healed. **(N–Q)** Gross changes in the tumor over time. **(R)** H&E staining image of samples taken from the abdominal nodule of the patient in case 4. **(S)** Very strong immunohistochemical staining (IHC 3+) of HER2 was observed in the samples taken from the abdominal nodule of the patient in case 4.

A 45-year-old woman was admitted to our hospital for evaluation on March 31, 2020 due to a left breast skin ulceration that had persisted for 8 months. Her whole left breast was enlarged and hard, covered with ulcers, nodules, and yellow–white pus, and the nipple–areola complex could not be clearly seen (**Figures 3A, L**); and her PS was 3. She had no smoking and drinking habits and no chronic diseases. We performed a biopsy of her nodule in the thoracic and abdominal wall on April 1, 2020 (the nodule is marked in **Figure 3A**). The examination results on other organs revealed that her lymph nodes located in mediastinum, bilateral axillary, and supraclavicular region all had metastasis. She also had significant liver metastases (**Figure 3N**). The pathological results showed that she had HER2-positive BC (**Figures 3R, S**). Based on these findings combined with NCCN (V2020.1), her tumor (T), lymph node (N), and metastasis (M) staging is cT4N4M1 (stage IV). We developed a regimen of albumin paclitaxel and trastuzumab combined with pyrotinib and capecitabine for her treatment. The interval of medication was adjusted according to the anemia and hypoproteinemia of the patient during treatment (**Supplementary Table S1** **)**. She received pyrotinib combined with capecitabine on April 9, 2020 and albumin paclitaxel combined with trastuzumab on April 11, 2020. During the 2 days of treatment with only pyrotinib and low-dose capecitabine, the redness and swelling that had spread to the lesions on the left rib area and the contralateral breast disappeared, and the swollen tissue shrunk to obvious wrinkles (**Figure 3B**). On the fifth day of treatment, the patient's tumor further shrunk (**Figure 3C**). On the 10th day of treatment, the tumor of the patient had shrunk significantly (**Figure 3D**). On the 13th day, the left breast had become flat, and the nipple wrapped in the tumor was exposed (**Figure 3E**). In subsequent treatment, the patient's tumor continued to shrink (**Figures 3F–K, M**). The symptoms of the patient continuously improved as the treatment continued, including a reduction in liver metastases (**Figures 3N–Q**). On April 20, 2021, her significant tumor masses had been completely replaced by new skin and her liver lesions had shrunk significantly (**Figure 3Q**). The last follow-up time was November 17, 2021, when she had been on treatment for 20 months.

### Case 5

**Figure 4:**
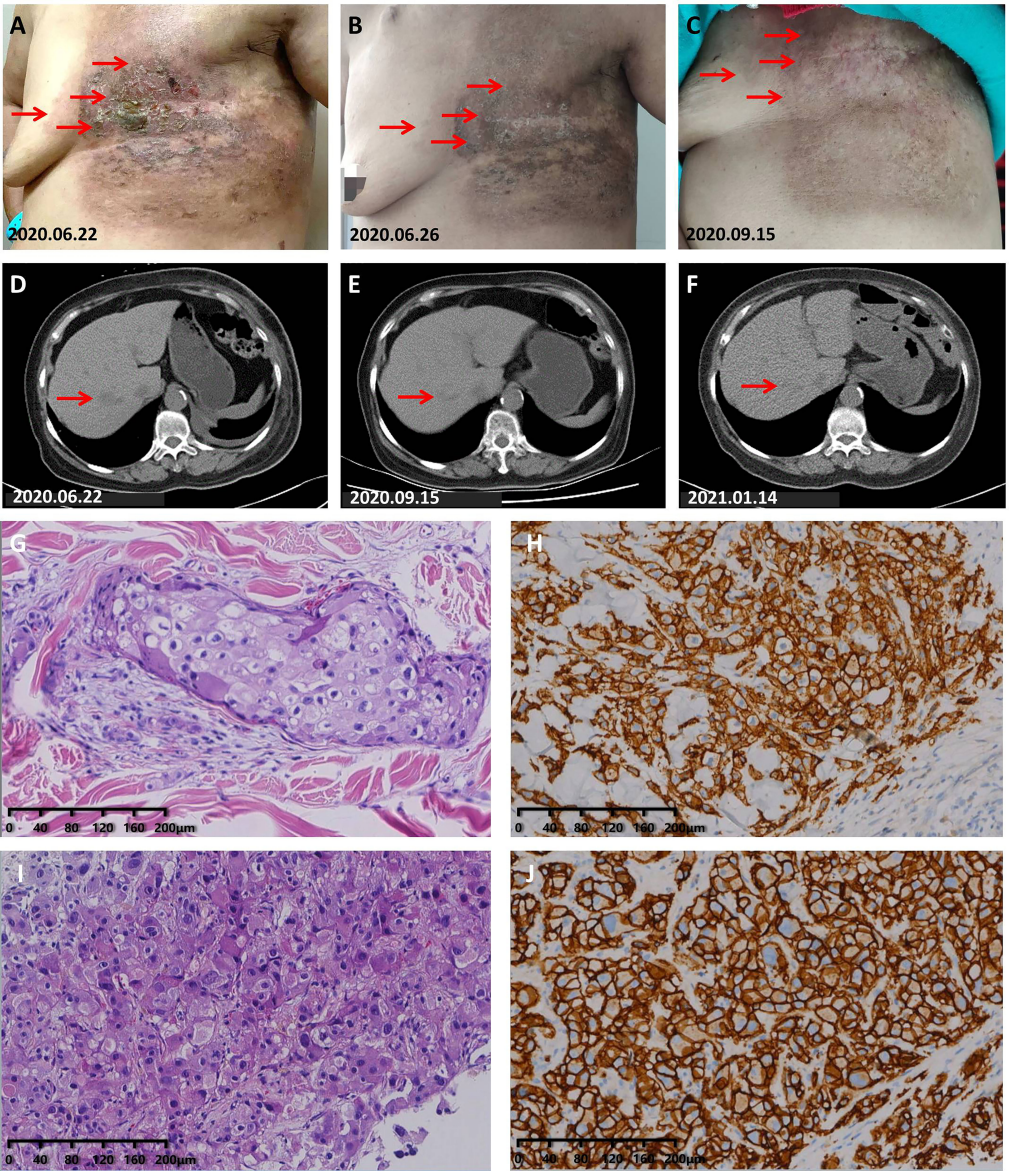
Partial response to capecitabine combined with trastuzumab and pyrotinib treatment. **(A)** Photograph taken before treatment: The skin on the left chest wall was diffused with erosion and exuding hard nodules, and the skin around the edge of the lesion was red and swollen. **(B)** Photograph taken on the 4th day during the 1st cycle of treatment: The exudation had completely disappeared, the erosional area had healed, and the redness and swelling of the skin had obviously subsided. **(C)** The skin on the left chest wall had completely healed. **(D)** Chest CT scans before treatment. **(E)** CT scans taken on September 15, 2020 showed that the liver lesions had shrunk significantly. **(F)** CT scans showed that the liver lesions had been further reduced. **(G)** H&E staining image of tumor samples taken from the liver of the patient in case 5. **(H)** Very strong immunohistochemical staining (IHC 3+) of HER2 was observed in the tumor samples taken from the liver of the patient in case 5. **(I)** H&E staining image of tumor samples taken from the left chest wall of the patient in case 5. **(J)** Very strong immunohistochemical staining (IHC 3+) of HER2 was observed in the tumor samples taken from the left chest wall of the patient in case 5.

A 64-year-old woman with type 2 diabetes came to our department on June 11, 2020 due to skin ulcers on the chest wall that did not heal after radiotherapy. The patient first attended our hospital on March 16, 2019 in the past, and her abnormal skin on the left chest wall appeared immediately after radiotherapy. The treatment process, the time of disease progression, and the factors that impact our decision-making process are shown in **Supplementary Figure S4**. After admission, we found that her left chest wall was diffused with erosions companied by exudation, and the surrounding skin was red and swollen (**Figure 4A**); her performance status was 2. She had no smoking and drinking habits and no chronic diseases other than type 2 diabetes mellitus. The results of a further examination revealed abnormal enlargement of the right axillary lymph nodes and a suspected tumor in the liver (**Figure 4D**). The results of a pathological examination demonstrated that her HER2-positive BC metastasized (**Figures 4G–J**). We confirmed that she had stage IV BC according to the NCCN Guidelines, and we treated her with capecitabine combined with trastuzumab and pyrotinib. Her cutaneous metastases quickly healed after receiving treatment (**Figures 4B, C**). Her liver metastases were also gradually shrinking during treatment (**Figure 4E**). An examination of the liver lesions in January 2021 revealed that the liver metastases had almost disappeared (**Figure 4F**). The last follow-up was on October 14, 2021. The follow-up results showed that her disease was well controlled when she had received more than 17 months of maintenance treatment.

## Discussion

In case 1, the progression of the cutaneous lesions of the patient during the taxotere, carboplatin, and herceptin treatment indicates that she is resistant to trastuzumab and may also exhibit systemic drug (such as pertuzumab and TDM-1) resistance (31). Clinical trials have confirmed that patients with HER2 overexpression can respond well to lapatinib (a reversible TKI for EGFR and HER2) after trastuzumab treatment fails (32). In addition, the phase II clinical trial of pyrotinib showed that the application of pyrotinib is more beneficial than the application of lapatinib in the population undergoing multi-line treatment (33). Previous studies found that pyrotinib can reverse the 5-fluorouracil resistance of HER2 BC cells (34), and there is evidence that HER2-targeting TKIs can modulate the ADCC response (35, 36). The rapid improvement of cutaneous metastases during the treatment of case 1 not only highlights the sensitivity to treatments containing pyrotinib but also suggests that the combination of drugs may have a synergistic effect on cell apoptosis. Thus, trastuzumab combined with pyrotinib and capecitabine may be an effective treatment for the HER2-positive MBC patients receiving multi-line treatment. Case 2 had a primary resistance to trastuzumab and taxane drugs, so we added pyrotinib and changed the chemotherapy drugs. Her cutaneous metastases disappeared after she received two courses of treatment, and her lung and intracranial metastases were also well controlled, even after drug withdrawal and re-treatment. Her disease was still under control during the period she only took pyrotinib irregularly, and that reminds us that pyrotinib may be appropriate as a second-line or even first-line therapy for HER2-positive MBC patients. At the time of consultation of case 3, the use of pertuzumab for advanced BC had not yet been approved for indications in China (the approval date was December 10, 2019), and TDM-1 had not yet been officially listed in China (the approval date was January 21, 2020). Therefore, we considered pyrotinib given its therapeutic effect on the cutaneous metastases of the first two cases and formulated a dual-targeted treatment plan of trastuzumab combined with pyrotinib. As predicted, her cutaneous metastases were quickly controlled, and this good treatment effect provided a basis for the formulation of the treatment plan for case 4.

Based on the poor condition of case 4, capecitabine was used in a gradually increasing method (from 600 mg/m^2^ of capecitabine) in the first course, and its dose was adjusted gradually according to the nutritional status of the patient until reaching the target dose. It is worth noting that the patient started to take pyrotinib and a small dose (600 mg/m^2^) of capecitabine on April 9, 2020. However, due to the superficial venous atrophy caused by malnutrition, the patient had difficulty with infusion. The use of albumin paclitaxel and trastuzumab was not initiated until the successful placement of the peripherally inserted central catheter on April 11, 2020. During 2 days of treatment with only pyrotinib and low-dose capecitabine, her cutaneous metastases had been quickly controlled. This suggests that single-agent pyrotinib may be also effective for patients with HER2-positive MBC who have not previously received trastuzumab therapy. In case 5, the patient had diabetes and was treated with insulin. Insulin and insulin-like growth factor-1 have been confirmed to be autocrine and paracrine mitogenic factors of BC cells and are considered to be possible mechanisms of targeted therapy resistance (37). The sensitivity of pyrotinib in case 5 may provide a new direction for basic research in HER2-positive MBC patients with diabetes.

With the extension of application time of HER2-targeted drugs, the drug resistance of targeted drugs has gradually attracted the attention of researchers. Several mechanisms of trastuzumab resistance have been extension (38), including changes in mucin 4 or cluster of differentiation-44 (CD44) expression (39, 40), NH2-terminally truncated form of human epidermal growth factor receptor 2 production (41), phosphatase and tensin homolog deleted on chromosome ten, or phosphatidylinositol 3 kinase mutations (42), and changes in ADCC (43). In the course of treatment of the patient in case 1, we found that the regimen containing pyrotinib seemed to have an amazingly rapid therapeutic effect on the cutaneous metastasis of HER2-positive BC. The treatment response in cases 2 and 3 reinforced this impression. Due to accidental factors, we excluded the effect of drugs other than low-dose capecitabine on the rapid effect of pyrotinib on cutaneous metastases in case 4, while patients in cases 2 and 3 were not treated with capecitabine. Considering these factors, we believe that the efficacy of pyrotinib in the treatment of HER2-positive MBC with cutaneous metastases is not based on other drugs and may be related to its own mechanism of action.

The molecular mechanism metastasis of BC cells to the skin remains unclear (44–48). A study in 2020 showed that the expression of Sialyl-Lewis x (SLe^x^) and glycosylated MUC1 (uMUC1) is associated with the incidence of BC skin metastasis (*p* < 0.05) (49). A high level of SLe^x^ expression has been confirmed to be associated with lymphatic vascular invasion in various tumors (50); MUC1 contains cancer-relevant immature O-glycans. Although extensive efforts have been made to develop anticancer antibodies targeting it, no anti-MUC1 antibody recognizes carbohydrates and the proximal MUC1 peptide region (51). MUC1 is a potential scaffold protein for SLe^x^ (50, 52), and its cytoplasmic tail (MUC1-C) interacts with many tyrosine kinase receptors, including EGFR and ErbB2 on the cell membrane. Studies have shown that erlotinib (a selective EGFR-TKI) can inhibit the growth of paclitaxel-resistant cervical cancer stem cells by blocking the EGFR-CREB (cAMP-response element binding protein)/glucocorticoid receptor β–interleukin- 6 axis in MUC1-positive cervical cancer (53). Pyrotinib is a reversible pan-ErbB receptor TKI that has inhibitory effects on EGFR/HER1, HER2, and HER4 (33). The results of these studies indicate that the pan-inhibitory effect of pyrotinib on ErbB receptors may prevent their interaction with MUC1-C, thus inhibiting the expression of MUC1. Therefore, pyrotinib has a dual role of anti-HER2 therapy and anti-MUC1 therapy in the cutaneous metastases of HER2-positive BC.

To the best of our knowledge, this is the only report demonstrating the fastest efficacy of pyrotinib in severe HER2-positive BC with cutaneous metastases as a near-monotherapy. These results are novel and provide a direction for further research. However, our hypothesis needs to be further verified, and relevant work is underway to collect similar cases for retrospective analysis.

## Conclusion

Pyrotinib has a rapid and significant therapeutic effect on the cutaneous metastasis of HER2-positive breast cancer. This effect is maintained even in trastuzumab-resistant patients. This therapeutic effect may be based on its dual role of anti-HER2 therapy and anti-MUC1 therapy in cutaneous metastases of HER2-positive breast cancer.

## Data Availability Statement

The original contributions presented in the study are included in the article/**Supplementary Material**. Further inquiries can be directed to the corresponding author.

## Ethics Statement

Written informed consent was obtained from the individual(s) for the publication of any potentially identifiable images or data included in this article.

## Author Contributions

NW, LL, and YX identified the case. JC, XL, FW, and CZ collected the clinical, diagnostic, and therapeutic information as well as images of the patients. NW wrote and submitted the manuscript. LL revised the manuscript. YG proofread the manuscript. All authors contributed to the article and approved the submitted version.

## Funding

Science and Technology Department of Henan Province; Henan Medical Science and Technology Joint Building Program (Award Number: LHGJ20200356); Recipient: NW.

## Conflict of Interest

The authors declare that the research was conducted in the absence of any commercial or financial relationships that could be construed as a potential conflict of interest.

## Publisher’s Note

All claims expressed in this article are solely those of the authors and do not necessarily represent those of their affiliated organizations, or those of the publisher, the editors and the reviewers. Any product that may be evaluated in this article, or claim that may be made by its manufacturer, is not guaranteed or endorsed by the publisher.
