# Supplementary material for: Case Report: Significant Efficacy of Pyrotinib in the Treatment of Extensive Human Epidermal Growth Factor Receptor 2-Positive Breast Cancer Cutaneous Metastases: A Report of Five Cases
Source: Front Oncol. 2021 Dec 16;11:729212. doi: 10.3389/fonc.2021.729212 (PMC8716402; doi:10.3389/fonc.2021.729212)
Supplement: Supplementary file 1 [file DataSheet_1.docx]

Supplementary Figure 1

**SUPPLEMENT FIGURE 1** Flowchart of the medical events and disease progression of case 1. **(A)** The post-surgery pathology immunohistochemistry indicated ER(−), PR(−), HER-2(3+). The treatment regimen comprised docetaxel 75 mg/m² IV day 1, doxorubicin 50 mg/m² IV day 1, and cyclophosphamide 500 mg/m² IV day 1, cycled every 21 days for 4 cycles. **(B)** The patient underwent the TAC regimen (intensified chemotherapy) three times during this period. **(C)** Trastuzumab 8 mg/kg IV week 1, followed by trastuzumab 6 mg/kg IV, cycled every 21 days. **(D)** The skin nodule that the patient found on her left chest wall was subsequently cut for pathological examination**. (E)** The pathological results showed that her breast cancer had invasion and metastases. Immunohistochemistry indicated ER(−), PR(−), HER-2(3+), Ki-67 (approximately 50% +). **(F)** The patient was treated with vinorelbine combined with S-1 (tegafur gimeracil oteracil potassium capsule); the specific usage and dosage are unknown. She then developed abnormal liver function and was hospitalized. **(G)** She was treated at another hospital with a “trastuzumab combined with docetaxel and carboplatin” regimen for 5 cycles. **(H)** During treatment, her radiographic evaluation was SD (Stable Disease), but her skin nodules progressed slowly and consistently. A photograph of her skin lesion is shown in **Figure 1(A^1^). (I)** A new mass was removed from the left chest wall for histopathological examination, which confirmed breast cancer progression. The immunohistochemical findings were ER(−), PR(−), HER-2(3+). **(J)** Trastuzumab 6 mg/kg IV, cycled every 21 days. Capecitabine 1250 mg/m^2^ twice daily on days 1–14, cycled every 21 days. Pyrotinib 400 mg once daily, days 1–21, cycled every 21 days. **(K)** A photograph of the skin lesion is shown in **Figure 1(B^1^)**.
