# Supplementary material for: Case Report: Significant Efficacy of Pyrotinib in the Treatment of Extensive Human Epidermal Growth Factor Receptor 2-Positive Breast Cancer Cutaneous Metastases: A Report of Five Cases
Source: Front Oncol. 2021 Dec 16;11:729212. doi: 10.3389/fonc.2021.729212 (PMC8716402; doi:10.3389/fonc.2021.729212)
Supplement: Supplementary file 2 [file DataSheet_2.docx]

Supplementary Figure 2

**SUPPLEMENT FIGURE 2** Flowchart showing the medical events and disease progression experienced by case 2. **(A, B)** The patient rejected our proposal (neoadjuvant therapy) and instead requested surgery. **(C)** After surgery, the patient received adjuvant chemotherapy (AC-T regimen), which comprised dose-dense AC followed by paclitaxel doxorubicin 60 mg/m^2^ IV day 1 and cyclophosphamide 600 mg/m^2^ IV day 1. The regimen was cycled every 14 days for 4 cycles and was followed by paclitaxel 175 mg/m^2^ in a 3 h IV infusion on day 1, cycled every 14 days for 4 cycles. **(D)** The patient refused trastuzumab treatment because trastuzumab was not included in China’s basic medical insurance reimbursement list in February 2017. **(E)** The patient accepted radiotherapy as planned. **(F)** Trastuzumab was included in the China Medical Insurance reimbursement list in September 2017, and at that time she began to use trastuzumab. **(G)** The patient had brain and skin metastases after she was treated with trastuzumab for seven cycles. She rejected our suggestion that she should use pyrotinib and continued treatment with the "trastuzumab combined with temozolomide" regimen at her local hospital. **(H)** The specific method and amount of medication were unknown**. (I)** We followed up by phone and learned that her chest CT examination at the local hospital revealed no progression, but it is not known whether the brain metastases continued to worsen. **(J)** In December 2018, we learned through telephone follow-up that the results of her re-examination at the local hospital showed enlarged intracranial lesions and lung tumor metastases, but she still refused to acccept treatment. **(K)** Refer to **Figure 1 (C^2^, G^2^)** for lung metastases from breast cancer**.** **(L) Refer to Figure 1 (A^2^)** for skin metastases from breast cancer. **(M)** Vinorelbine (25 mg/m^2^, intravenously, on days 1, 8, and 15) every 21 days; trastuzumab 6 mg/kg IV, cycled every 21 days; and pyrotinib 400 mg once daily, days 1–21, cycled every 21 days. **(N)** Refer to **Figure 1 (B^2^, D^2^, H^2^)** for her rapidly shrinking lesions. **(O)** She chose to discontinue pyrotinib. **(P)** Refer to **Figure 1 (E^2^)** for the enlarged brain metastatic lesion. **(Q)** Refer to **Figure 1 (I^2^)** for the enlarged lung metastatic lesion. **(R)** Refer to **Figure 1 (F^2^)** for the reduced brain metastatic lesion. **(S)** Refer to **Figure 1 (J^2^)** for the reduced lung metastatic lesion**.**
