# Supplementary material for: Case Report: Significant Efficacy of Pyrotinib in the Treatment of Extensive Human Epidermal Growth Factor Receptor 2-Positive Breast Cancer Cutaneous Metastases: A Report of Five Cases
Source: Front Oncol. 2021 Dec 16;11:729212. doi: 10.3389/fonc.2021.729212 (PMC8716402; doi:10.3389/fonc.2021.729212)
Supplement: Supplementary file 3 [file DataSheet_3.docx]

Supplementary Figure 3

**SUPPLEMENT FIGURE 3** The process of diagnosis and treatment of case 3 and the time of disease progression. **(A)** The histopathological results in a local hospital indicated invasive cancer without clear immunohistochemical results. **(B)** Four chemotherapy sessions were performed after the surgery, but the specific drug category, usage, and dosage were unknown. **(C)** The patient was treated with letrozole tablets without knowing her hormone receptor was positive or negative. **(D)** The patient’s skin nodule that she found on her right chest wall was removed for pathological examination. **(E, F)** The pathological result was reported as breast cancer infiltration in the fibrous tissue of her right chest wall without being examined immunohistochemistry, and the specific dose of radiation therapy she received was not recorded. **(G)** She underwent further treatment with exemestane without knowing whether her hormone receptor was positive. **(H)** No treatment for the ulcerated skin lesions and the edematous upper limbs. **(I)** She continued exemestane orally, although her symptoms progressed. **(J)** At this time, she finally underwent immunohistochemical examination, the results of which showed ER(−), PR(−), HER-2(2+), Ki-67(20%+). Her HER2 status was not examined by FISH (Fluorescence In Situ Hybridization). **(K)** Docetaxel 75 mg/m^2^ IV day 1, cycled every 21 days for 6 cycles; pyrotinib 400 mg once daily, days 1–21, cycled every 21 days; and trastuzumab 8 mg/kg IV week 1, followed by trastuzumab 6 mg/kg IV, cycled every 21 days. **(L)** A photograph of the skin lesion is shown in **Figure 2B**. **(M)** A photograph of the skin lesion is shown in **Figure 2E.** **(N)** The patient chose to reduce the dosage of pyrotinib by half (200 mg once daily) as she reasoned that she was near the end of her treatment. **(O)** A photograph of the skin lesion is shown in **Figure 2F**. **(P)** Pyrotinib 400 mg once daily, days 1–21, cycled every 21 days; trastuzumab 6 mg/kg IV week 1, cycled every 21 days; and capecitabine 1250 mg/m^2^ twice daily on days 1–14, cycled every 21 days. She had completed six courses of chemotherapy and restored pyrotinib to the standard dosage. **(Q)** Refer to **Figure 2G** for images of nearly healed skin lesions.
