# Supplementary material for: Case Report: Significant Efficacy of Pyrotinib in the Treatment of Extensive Human Epidermal Growth Factor Receptor 2-Positive Breast Cancer Cutaneous Metastases: A Report of Five Cases
Source: Front Oncol. 2021 Dec 16;11:729212. doi: 10.3389/fonc.2021.729212 (PMC8716402; doi:10.3389/fonc.2021.729212)
Supplement: Supplementary file 4 [file DataSheet_4.docx]

Supplementary Figure 4

**SUPPLEMENT FIGURE 4**  The process of diagnosis and treatment of case 5 and the time of her disease progression. **(A)** Pathological examination of the left breast tumor and left axillary enlarged lymph node puncture tissue to obtain the results of breast invasive carcinoma. The immunohistochemical status was: ER(−), PR(−), HER-2(3+), Ki-67(60%+). **(B)** Pertuzumab was first approved by the NMPA (National Medical Products Administration) on December 17, 2018 as an adjuvant therapy for patients with early HER-2+ breast cancer with a high risk of recurrence; its indications for neoadjuvant therapy for patients with HER-2+, locally advanced, inflammatory or early breast cancer (diameter > 2 cm or lymph node positive) were approved on August 16, 2019. **(C)** Doxorubicin 60 mg/m^2^ IV day 1, cyclophosphamide 600 mg/m^2^ IV day 1, cycled every 21 days for 4 cycles. **(D)** No changes in the size and hardness of the mass were found by palpation. **(E)** Docetaxel 75 mg/m^2^ IV day 1, carboplatin AUC 6 IV day 1, cycled every 21 days for 6 cycles; and trastuzumab 8 mg/kg IV week 1, followed by trastuzumab 6 mg/kg IV, cycled every 21 days. Pertuzumab was approved as a first-line treatment for HER2-positive advanced breast cancer in China on December 10, 2019. **(F)** TDM-1 was approved on January 22, 2020 for the treatment of HER2-positive early breast cancer with aggressive lesions remaining after completion of neoadjuvant therapy based on taxanes combined with trastuzumab. **(G)** The patient rejected the dual-targeted therapy recommendation of trastuzumab combined with tyrosine kinase inhibitors, and accepted trastuzumab treatment only. **(H)** The patient could not accurately describe when the abnormal symptoms appeared because of the presence of skin thickening and edema after radiotherapy. **(I)** She was treated with topical ointment containing antibiotics and traditional Chinese medicine. **(J)** Liver tissue puncture was pathologically diagnosed as breast cancer metastasis. Immunohistochemistry results were: ER(−), PR(−), HER-2(3+), Ki-67(30%+). **(K)** The tissue punctured from the enlarged right axillary lymph node was pathologically diagnosed as breast cancer metastasis. The immunohistochemistry results were as follows: ER(−), PR(30% weak +), HER-2(3+), Ki-67 (30%+). **(L)** The tissue obtained by cutting the hard nodules of the skin on the left chest wall was used for pathological examination and was diagnosed as breast cancer metastatic lesions. The immunohistochemical results were as follows: ER(−), PR(−), HER-2(3+), Ki-67(30%+). **(M)** Trastuzumab 6 mg/kg IV, cycled every 21 days; capecitabine 1000 mg/m^2^ twice daily on days 1–14, cycled every 21 days; and pyrotinib 400 mg once daily, days 1–21, cycled every 21 days. **(N)** As shown in **Figure 4(C)**. **(O)** As shown in **Figure 4(E)**.

*The patient did not achieve significant tumor regression during treatment with anthracyclines; the histopathological assessment of Miller-Payne after the application of the TCH regimen was grade II, and her disease progression occurred during maintenance therapy with trastuzumab. These findings suggest multiple resistance to trastuzumab, paclitaxel, and anthracyclines.

* Her diarrhea symptoms during the treatment were relieved after adjusting the diet and applying montmorillonite powder, and the treatment and had no effect on her quality of life. She developed grade 3 hand-foot syndrome characterized by peeling, pain, and swelling of the extremities, which may be related to the drug effects of capecitabine. These symptoms were somewhat relieved by topical application of urea cream and carbomer.
