# Supplementary material for: Case Report: Significant Efficacy of Pyrotinib in the Treatment of Extensive Human Epidermal Growth Factor Receptor 2-Positive Breast Cancer Cutaneous Metastases: A Report of Five Cases
Source: Front Oncol. 2021 Dec 16;11:729212. doi: 10.3389/fonc.2021.729212 (PMC8716402; doi:10.3389/fonc.2021.729212)
Supplement: Supplementary file 5 [file DataSheet_5.docx]

Supplementary Material 1

# Specific Description of Chemotherapy Protocol Abbreviation in NCCN Guidelines

**●AC followed by docetaxel + trastuzumab**

▲Doxorubicin 60 mg/m^2^ IV day 1

▲Cyclophosphamide 600 mg/m^2^ IV day 1

◊ Cycled every 21 days for 4 cycles

◊ Followed by:

▲Docetaxel 100 mg/m^2^ IV day 1

◊ Cycled every 21 days for 4 cycles

◊ With:

▲Trastuzumab 4 mg/kg IV wk 1

◊ Followed by:

▲ Trastuzumab 2 mg/kg IV weekly for 11 wks

◊ Followed by:

▲Trastuzumab 6 mg/kg IV

◊ Cycled every 21 days to complete 1 y of trastuzumab therapy.

●AC followed by docetaxel + trastuzumab + pertuzumab

▲Doxorubicin 60 mg/m^2^ IV day 1

▲ Cyclophosphamide 600 mg/m^2^ IV day 1

◊ Cycled every 21 days for 4 cycles

◊ Followed by:

▲Pertuzumab 840 mg IV day 1 followed by 420 mg IV

▲Trastuzumab 8 mg/kg IV day 1 followed by 6 mg/kg IV

▲Docetaxel 75–100 mg/m^2^ IV day 1

◊ Cycled every 21 days for 4 cycles

◊ Followed by:

▲Trastuzumab 6 mg/kg IV

▲Pertuzumab 420 mg IV day 1

◊ Cycled every 21 days to complete 1 y of therapy.

●TCH

▲Docetaxel 75 mg/m^2^ IV day 1

▲Carboplatin AUC 6 IV day 1

◊ Cycled every 21 days for 6 cycles

◊ With:

▲Trastuzumab 4 mg/kg IV wk 1

◊ Followed by:

▲Trastuzumab 2 mg/kg IV for 17 wks

◊ Followed by:

▲ Trastuzumab 6 mg/kg IV

◊ Cycled every 21 days to complete 1 y of therapy.

OR

▲Trastuzumab 8 mg/kg IV wk 1

◊ Followed by:

▲Trastuzumab 6 mg/kg IV

◊ Cycled every 21 days to complete 1 y of therapy.

●TCH + pertuzumab

▲Docetaxel 75 mg/m^2^ IV day 1

▲Carboplatin AUC 6 IV day 1

◊ Cycled every 21 days for 6 cycles

With:

▲Trastuzumab 8 mg/kg IV day 1

▲ Pertuzumab 840 mg IV day 1

◊ Followed by:

▲ Trastuzumab 6 mg/kg IV on day 1

▲Pertuzumab 420 mg IV day 1

◊ Cycled every 21 days to complete 1 y of therapy.

●Paclitaxel + trastuzumab

▲ Paclitaxel 80 mg/m^2^ IV weekly for 12 weeks

◊ With:

▲Trastuzumab 4 mg/kg IV with first dose of paclitaxel

◊ Followed by:

▲Trastuzumab 2 mg/kg IV weekly to complete 1 y of treatment. As an alternative, trastuzumab 6 mg/kg IV every 21 days may be used following the completion of paclitaxel, and given to complete 1 y of trastuzumab treatment.

●AC followed by T + trastuzumab

▲Doxorubicin 60 mg/m^2^ IV day 1

▲Cyclophosphamide 600 mg/m^2^ IV day 1

◊ Cycled every 21 days for 4 cycles.

◊ Followed by:

▲Paclitaxel 80 mg/m^2^ by 1 h IV weekly for 12 wks

◊ With:

▲ Trastuzumab 4 mg/kg IV with first dose of paclitaxel

◊ Followed by:

▲Trastuzumab 2 mg/kg IV weekly to complete 1 y of treatment. As an alternative, trastuzumab 6 mg/kg IV every 21 days may be used following the completion of paclitaxel, and given to complete 1 y of trastuzumab treatment.

●Dose-dense AC followed by paclitaxel + trastuzumab

▲Doxorubicin 60 mg/m^2^ IV day 1

▲Cyclophosphamide 600 mg/m2 IV day 1

◊ Cycled every 14 days for 4 cycles.

◊ Followed by:

▲ Paclitaxel 175 mg/m^2^ by 3 h IV infusion day 1

◊ Cycled every 14 days for 4 cycles.

◊ With:

▲ Trastuzumab 4 mg/kg IV with first dose of paclitaxel

◊ Followed by:

▲Trastuzumab 2 mg/kg IV weekly to complete 1 y of treatment. As an alternative, trastuzumab 6 mg/kg IV every 21 days may be used following the completion of paclitaxel, and given to complete 1 y of trastuzumab treatment.

●Trastuzumab + vinorelbine

▲Vinorelbine

◊ 25 mg/m2 IV day 1 weekly; or

◊ 20–35 mg/m2 IV days 1 and 8; cycled every 21 days; or

◊ 25–30 mg/m2 IV days 1, 8, and 15; cycled every 28 days

▲Trastuzumab 4 mg/kg IV day 1 followed by 2 mg/kg IV weekly

Or

▲Trastuzumab 8 mg/kg IV day 1 followed by 6 mg/kg IV day 1 every 21 days

● Trastuzumab + capecitabine

▲Capecitabine 1000–1250 mg/m^2^ PO twice daily days 1–14 cycled every 21 days

▲Trastuzumab 4 mg/kg IV day 1 followed by 2 mg/kg IV weekly

Or

▲Trastuzumab 8 mg/kg IV day 1 followed by 6 mg/kg IV day 1 every 21 days
