# Supplementary material for: Case Report: Significant Efficacy of Pyrotinib in the Treatment of Extensive Human Epidermal Growth Factor Receptor 2-Positive Breast Cancer Cutaneous Metastases: A Report of Five Cases
Source: Front Oncol. 2021 Dec 16;11:729212. doi: 10.3389/fonc.2021.729212 (PMC8716402; doi:10.3389/fonc.2021.729212)
Supplement: Supplementary file 6 [file DataSheet_6.docx]

Supplementary Material 2

**Abbreviations**

BC, Breast Cancer; EGFR, epidermal growth factor receptor; HER1, human epidermal growth factor receptor 1; HER2, human epidermal growth factor receptor 2; HER4, human epidermal growth factor receptor 4; mAb, monoclonal antibodies; ER, estrogen receptor; PR, progesterone receptor; HR, hormone receptor; TNBC, Triple Negative Breast Cancer; MBC, Advanced Breast Cancer; ADCC, antibody-mediated cell-dependent cytotoxicity; TKR, tyrosine kinase receptors; TKI, tyrosine kinase inhibitors; ICD, intracellular domain; ECD, extracellular domain; PFS, Progression Free Survival; PICC, Peripherally Inserted Central Catheter; T-DM1, Trastuzumab emtansine; DS-8201, trastuzumab deruxtecan; MARGENZA, margetuximab-cmkb; ErbB, Erythroblastic Leukemia Viral Oncogene Homolog; MAPK, mitogen-activated protein kinase; PI-3K, phosphatidylinositol 3 kinase; AKT, protein kinase B; MUC4, mucin-4; PTEN, Phosphatase and tensin homologue deleted on chromosome 10; PI3K, phosphatidylinositol 3-kinase; TP53, tumor protein p53.
