# Supplementary figures and images for: Case Report: Significant Efficacy of Pyrotinib in the Treatment of Extensive Human Epidermal Growth Factor Receptor 2-Positive Breast Cancer Cutaneous Metastases: A Report of Five Cases
Source: Front Oncol. 2021 Dec 16;11:729212. doi: 10.3389/fonc.2021.729212 (PMC8716402; doi:10.3389/fonc.2021.729212)

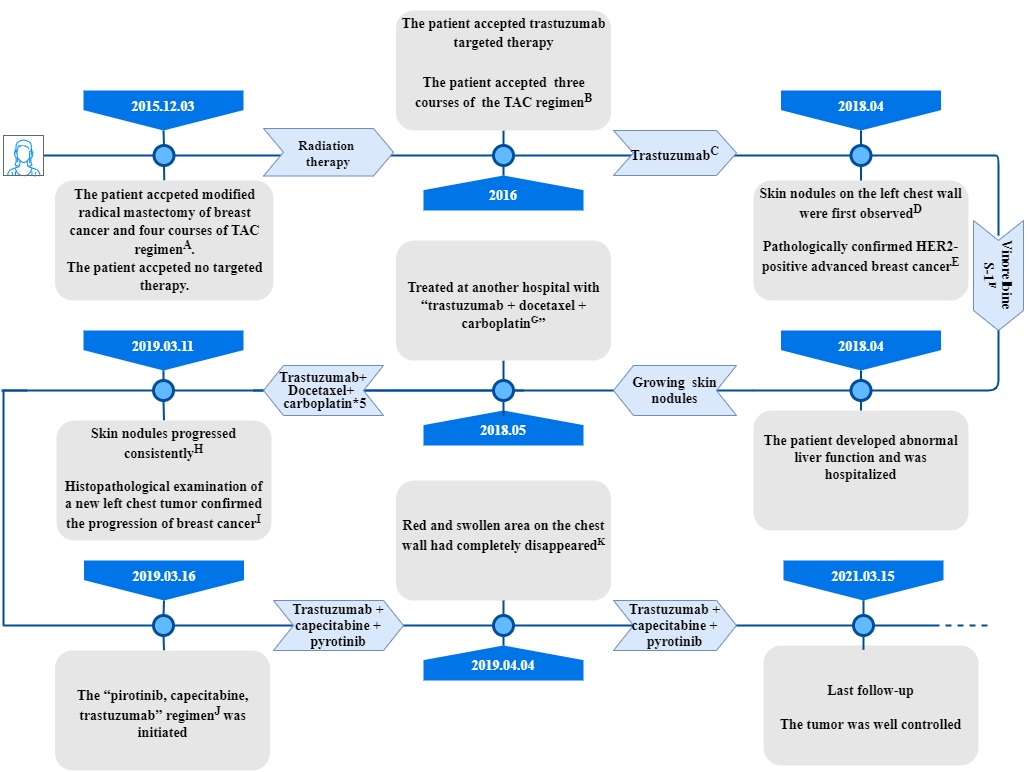

Supplement: Supplementary file 7 [file Image_1.jpeg]

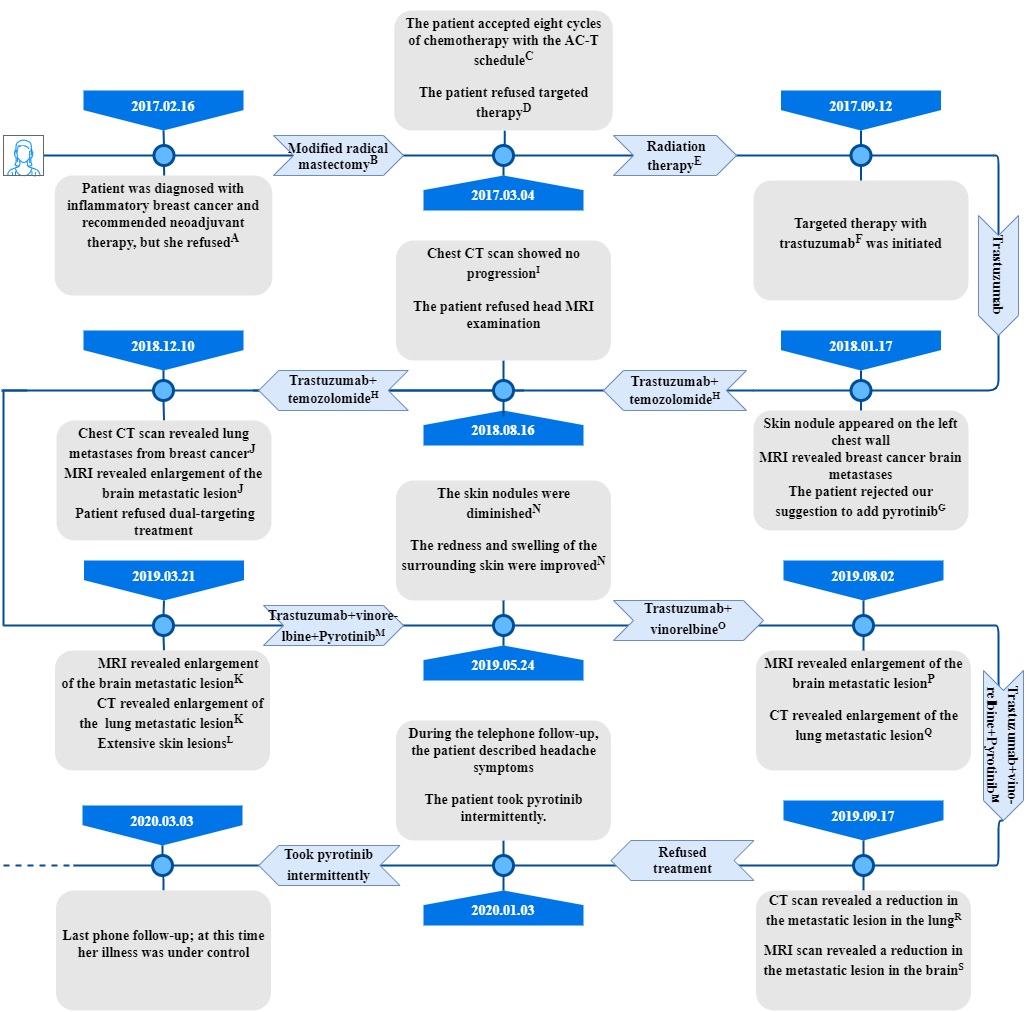

Supplement: Supplementary file 8 [file Image_2.jpeg]

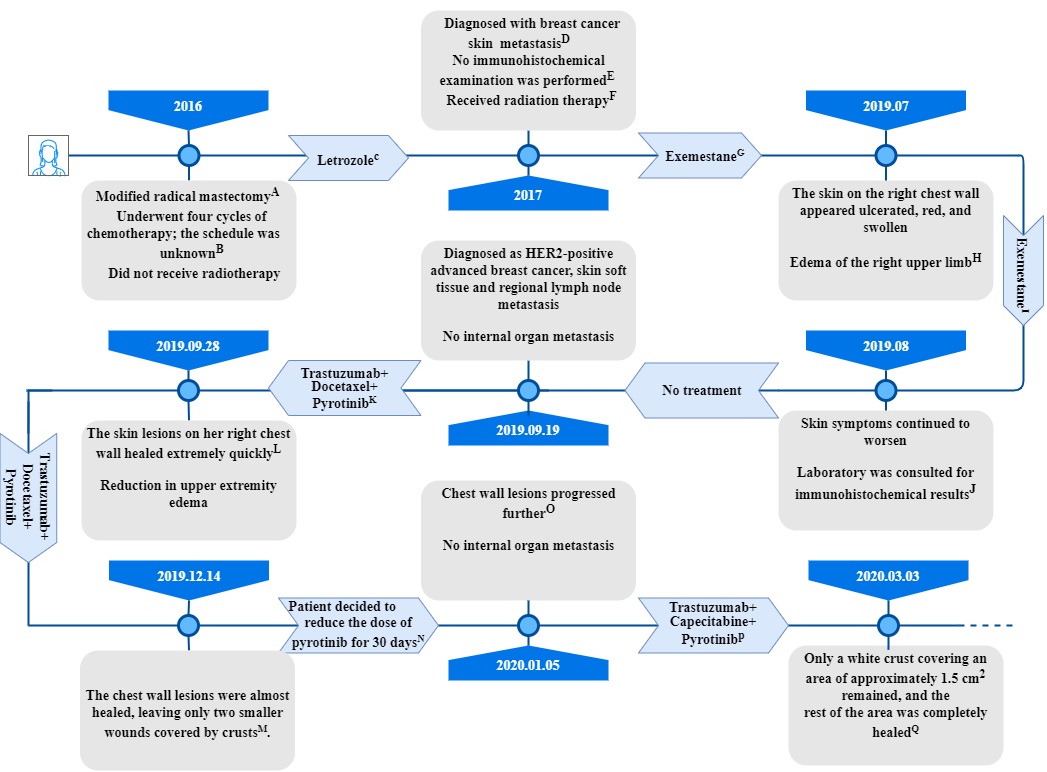

Supplement: Supplementary file 9 [file Image_3.jpeg]

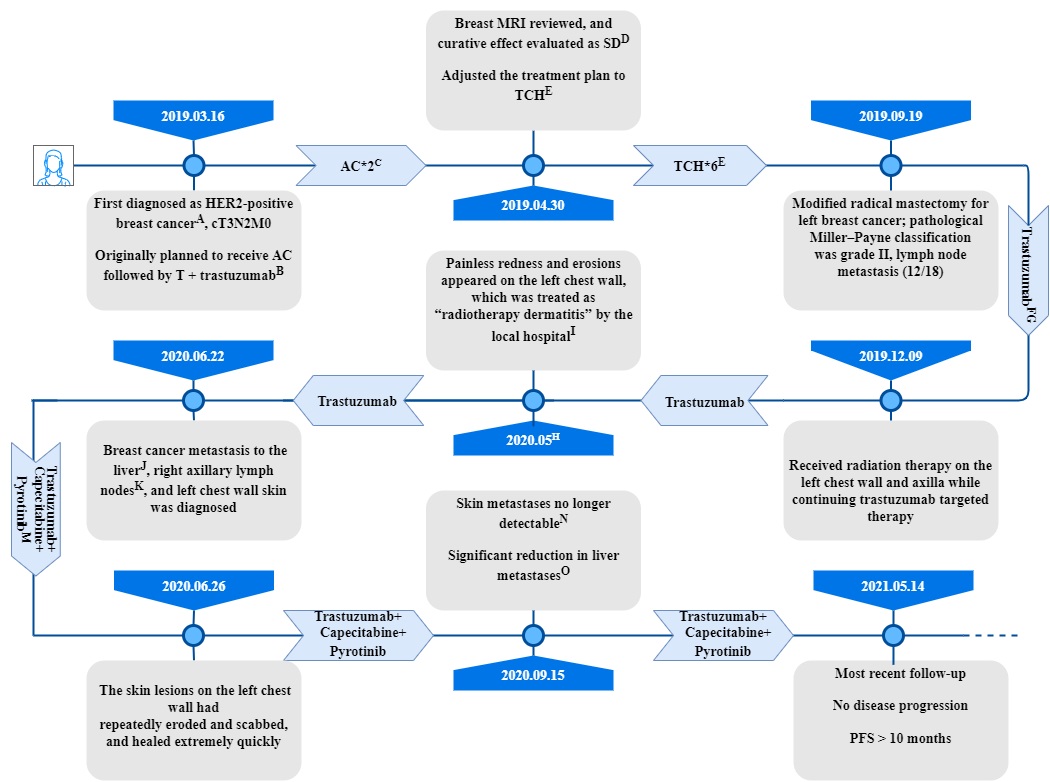

Supplement: Supplementary file 10 [file Image_4.jpeg]
