# Supplementary material for: Case Report: Significant Efficacy of Pyrotinib in the Treatment of Extensive Human Epidermal Growth Factor Receptor 2-Positive Breast Cancer Cutaneous Metastases: A Report of Five Cases
Source: Front Oncol. 2021 Dec 16;11:729212. doi: 10.3389/fonc.2021.729212 (PMC8716402; doi:10.3389/fonc.2021.729212)
Supplement: Supplementary file 11 [file Table_1.docx]

Supplementary Table 1

**SUPPLEMENTARY TABLE 1** The presence of anemia and hypoproteinemia in Case 4 prevented the patient from following the strict weekly chemotherapy schedule. Her chemotherapy interval was therefore longer than the standard weekly chemotherapy regimen.

| **Medication situation and time** | | |
| --- | --- | --- |
| **Course of treatment** | **Start date** | **Treatment used** |
| 1st | 2020.04.09 | Capecitabine + trastuzumab + pyrotinib + albumin paclitaxel^a^ |
| 2nd | 2020.04.20 | Capecitabine + pyrotinib + albumin paclitaxel^b^ |
| 3rd | 2020.05.09 | Capecitabine + trastuzumab + pyrotinib + albumin paclitaxel^c^ |
| 4th | 2020.05.19 | Pyrotinib + albumin paclitaxel^d^ |
| 5th | 2020.05.28 | Capecitabine + trastuzumab + pyrotinib + albumin paclitaxel^c^ |
| 6th | 2020.06.12 | Capecitabine + pyrotinib + albumin paclitaxel^e^ |
| 7th | 2020.06.19 | Trastuzumab + pyrotinib + albumin paclitaxel^f^ |
| 8th | 2020.06.26 | Capecitabine + pyrotinib + albumin paclitaxel^e^ |
| 9th | 2020.07.10 | Trastuzumab + pyrotinib + albumin paclitaxel^f^ |
| 10th | 2020.07.17 | Capecitabine + pyrotinib + albumin paclitaxel^e^ |
| 11th | 2020.07.24 | Capecitabine + pyrotinib + albumin paclitaxel^e^ |
| 12th | 2020.07.31 | Trastuzumab + pyrotinib + albumin paclitaxel^f^ |
| Maintenance treatment | 2020.08.01 | Trastuzumab + pyrotinib + capecitabine^g^ |

*^a^Pyrotinib 400 mg once daily, days 1–21, cycled every 21 days; capecitabine 800 mg/m^2^ twice daily on days 1–14, cycled every 21 days (2020.04.09); albumin paclitaxel 150 mg/m^2^, IV, once a week; and trastuzumab 8 mg/kg IV week 1, followed by trastuzumab 6 mg/kg IV, cycled every 21 days (2020.04.11).*

*^b^Pyrotinib 400 mg once daily, days 1–21, cycled every 21 days; capecitabine 800 mg/m^2^ twice daily on days 1–14, cycled every 21 days; and albumin paclitaxel 150 mg/m^2^, IV, once a week.*

*^c^Pyrotinib 400 mg once daily, days 1–21, cycled every 21 days; capecitabine 1000 mg/m^2^ twice daily on days 1–14, cycled every 21 days; albumin paclitaxel 150 mg/m^2^, IV, once a week; and trastuzumab 6 mg/kg IV, cycled every 21 days.*

*^d^Pyrotinib 400 mg once daily, days 1–21, cycled every 21 days, and albumin paclitaxel 150 mg/m^2^, IV, once a week.*

*^e^Pyrotinib 400 mg once daily, days 1–21, cycled every 21 days; capecitabine 1000 mg/m^2^ twice daily on days 1–14, cycled every 21 days; and albumin paclitaxel 150 mg/m^2^, IV, once a week.*

*^f^Pyrotinib 400 mg once daily, days 1–21, cycled every 21 days; albumin paclitaxel 150 mg/m^2^, IV, once a week; and trastuzumab 6 mg/kg IV, cycled every 21 days.*

*^g^Pyrotinib 400 mg once daily, days 1–21, cycled every 21 days; capecitabine 1250 mg/m^2^ twice daily on days 1–14, cycled every 21 days; and trastuzumab 6 mg/kg IV, cycled every 21 days.*
